# Viewing angle matters in British Sign Language processing

**DOI:** 10.1038/s41598-024-51330-1

**Published:** 2024-01-10

**Authors:** Freya Watkins, Diar Abdlkarim, Bodo Winter, Robin L. Thompson

**Affiliations:** 1 School of Psychology, University of Birmingham, Edgbaston, Birmingham, UK; 2 Department of English Language and Linguistics, University of Birmingham, Edgbaston, Birmingham, UK

**Keywords:** Human behaviour, Psychology

## Abstract

The impact of adverse listening conditions on spoken language perception is well established, but the role of suboptimal viewing conditions on signed language processing is less clear. Viewing angle, i.e. the physical orientation of a perceiver relative to a signer, varies in many everyday deaf community settings for L1 signers and may impact comprehension. Further, processing from various viewing angles may be more difficult for late L2 learners of a signed language, with less variation in sign input while learning. Using a semantic decision task in a distance priming paradigm, we show that British Sign Language signers are slower and less accurate to comprehend signs shown from side viewing angles, with L2 learners in particular making disproportionately more errors when viewing signs from side angles. We also investigated how individual differences in mental rotation ability modulate processing signs from different angles. Speed and accuracy on the BSL task correlated with mental rotation ability, suggesting that signers may mentally represent signs from a frontal view, and use mental rotation to process signs from other viewing angles. Our results extend the literature on viewpoint specificity in visual recognition to linguistic stimuli. The data suggests that L2 signed language learners should maximise their exposure to diverse signed language input, both in terms of viewing angle and other difficult viewing conditions to maximise comprehension.

## Introduction

### Rationale

Various adverse conditions can interfere with language comprehension. In the case of spoken languages, adverse listening conditions involve linguistic factors like variable or ambiguous speech, and extralinguistic factors like background noise, e.g. when trying to talk near a loud train (for review, see e.g.^1^). Languages in the visuospatial modality, like signed languages, are produced in three-dimensional space by the hands, head and upper body, and perceived with the eyes. In this modality, adverse viewing conditions include similar linguistic factors like variable signing, but also unique extralinguistic factors like an impeded line of vision, or blurry video quality. A range of studies have examined how *linguistic* visuospatial features of signed languages, e.g. visual phonology and spatial morphology, affect comprehension (see e.g.^2–4^ for review). However, *extralinguistic* viewing conditions for signing, i.e. the visuospatial relationships between the producer, the receiver, and the environment, which have no straightforward analogues in spoken languages, are relatively under-explored and might also impact signed language processing.

One everyday context in which extralinguistic viewing conditions for sign comprehension can vary is the angle of orientation of the perceiver relative to the person signing (hereafter: ‘viewing angle’). If different viewing angles impact sign comprehension, there are implications for theories of how signs are represented in the mental lexicon, how signers deal with suboptimal input, and how signed languages are best taught and learned. Furthermore, viewing angle offers an opportunity to rethink modality-specific models of adverse conditions on spoken language processing and instead create models that account for effects of linguistic and extralinguistic variation in input on multimodal language processing more broadly.

The present study explores, for the first time, semantic processing of British Sign Language (BSL) signs presented from five different viewing angles. We study four groups of BSL signers with varying language backgrounds: deaf and hearing L1 signers, plus hearing L2 signers of advanced and intermediate proficiency, who have a spoken language as L1. First, we explore the background literature on adverse viewing conditions for signed language processing and the importance of viewing angle in signed languages, as well as effects of viewing angle in other processing contexts. We present the methodology and results of our distance priming paradigm, showing how BSL signers process signs from various viewing angles, firstly without any priming and then after being primed by a specific viewing angle. Sub-analyses also explore the role of mental rotation ability and sign type, i.e. whether a sign is articulated with one or two hands and whether it is symmetrical or not, in the context of viewing angle.

### Adverse viewing conditions for signed language

In spoken language research, a wide range of studies have investigated speech perception under ‘adverse conditions’, using both stimuli degraded artificially in the lab (for discussion, see e.g.^5^), as well as many kinds of more naturalistic variation that may impact listening comprehension. For example, research has examined the effects of background noise when communicating in public, unfamiliar accents, and poor listener working memory on L1 speech comprehension^1^. While visual information is also important in speech processing (see e.g.^6^, it is even more crucial in sign language processing. Despite this, there is relatively little work explicitly addressing adverse viewing conditions in signed language processing. Most work invoking adverse conditions has taken the former approach, employing less realistic stimuli to test the limits of sign intelligibility or minimal pair discrimination. Examples include adding artificial visual noise^7,8^ or reducing input via point-light displays^9–11^. Other studies have altered the environment, e.g. impeding visibility with barriers^12^, or limited the receiver’s perception by using goggles or blindfolds to prevent self-monitoring^13^. Aside from research on the intelligibility of signed language videos on early mobile phones with poor quality^14^, the aforementioned lab-generated stimuli and scenarios are somewhat artificial, telling us more about visual processing than everyday challenges of real-life signed language comprehension. Signed language corpus linguistics and sociolinguistics have described naturalistic variation in viewing conditions arising at the source, i.e. during production, such as visual foreign accent^15^ and regional variation^16^, but there is little work exploring the effects of such variation on perception. Here, we explore the extent to which differences in viewing angle, an environmental variation in viewing condition that occurs frequently, can impact signed language processing.

### Viewing angle in signed language

Work in Deaf Studies and deaf geographies have examined the proxemics, i.e. the human use of interpersonal space^17^, for signed language communication and how these relate to deaf culture (see e.g.^18–21^). Pairs of signers in dialogue usually position themselves opposite each other, since this best facilitates eye contact. Eye contact is important as the eyes are the resting gaze location for L1 signed language perception^22^, while a break in eye contact is one of several ways to signal desire for a conversational turn^23^. Sign language interpreters also need to consider proxemics, ensuring they are positioned so that deaf people can make eye contact with both the interpreter and any speakers^24^, which usually entails a frontal view of signing. Indeed, face-to-face views of signers, with a 0° viewing angle, are considered the “normal viewing conditions” for signing^25^. Despite this, there are many other everyday contexts where pairs of signers are not positioned face-to-face. For example, while walking along a street or driving in a car, signers need to attend to the environment and cannot fully turn to face each other while conversing^26^. Settings with ‘hearing architecture’, where seating is in fixed rows, e.g. lecture theatres or cinemas, can also force side views of signing. Positioning is sometimes negotiated and reconfigured if viewing conditions are difficult^27^, though not always. In group settings, interlocutors spend most of the time watching signing from various degrees of side angle (e.g., when sat in a circle). Deaf signers try to optimise viewing conditions by adapting their positions for different group sizes, e.g. by forming a triangle for three people; a diamond for four, etc. This strategy, named “deaf visual accommodation” by Bahan^18^ [p. 89], equalises lines of sight across the group, sharing and minimising the burden of adverse viewing angles. Thus, forming a “conversation circle” is common^19,28^. Indeed, for deaf signers this inclusive proxemic strategy is “embodied habitus”, i.e. typical of how deaf bodies use space^29^. However, it should be noted that such accommodation strategies are not always possible or simply not enacted in casual interactions, meaning a large amount of communication takes place at suboptimal viewing angles. Furthermore, if a viewer is not included as an active participant in a conversation but is instead ‘over-seeing’, or is watching remotely, viewing angles often become more extreme, closer to 90° than 0°. Thus, despite a clear preference to avoid them, side viewing angles are not unique, but everyday occurrences which are sometimes responded to by proxemic behaviours.

### L1 versus L2 exposure to adverse viewing angles

Existing studies featuring adverse viewing conditions for signed languages almost exclusively focus on L1 processing effects. Adverse listening conditions have been shown to disproportionately affect L2 listeners during speech processing^30^. Therefore, if side angles are an adverse viewing condition for sign processing, it is likely that the impact would be more severe on L2 sign learners compared to L1 signers. Viewing angle is a type of viewing condition where the relative frequency of exposure to different angles likely varies by language background. A child learning a signed language as L1 will receive sign input from all possible angles: while child-directed signing will mostly be seen from a frontal angle and from above, children will also view signs from the side during incidental learning. As L1 signers become active in the deaf community, they will frequently be in social situations involving conversation circles. Hearing L2 learners, on the other hand, tend to know fewer deaf people and often receive input primarily from a single frontal source at a time, e.g. an individual teacher, in dialogue with a deaf colleague or family member, or online teaching videos. Therefore, the proportion of face-on signed language input is likely to be higher for hearing L2 learners, with very little exposure to other viewing angles-possibly only from other learners in class. Even when attending deaf social events, hearing L2 learners struggle to follow rapid turn-taking and overlapping cross-talk, with greater problems the larger the discussion group^31^.

Deaf people, however, due to a larger visual field^32^, can usually see more than one person while signing to a group^33^ and are familiar with group turn-taking conventions. The differences between L1 and hearing L2 signers in exposure to signed language input from front versus side angles may lead to differences in how each group represents signs in the mental lexicon, and how they process ‘suboptimal’ input due to adverse viewing conditions. Since viewing angle can provide wider insights about how we encode and access mental representations, we now turn to look at the role of viewing angle in similar processing contexts.

### Viewing angle in action recognition

As in signed language processing, recognising human motion requires identifying 3D configurations of motor movements from 2D retinal images of a pose. Since this is a complex task, researchers have asked whether the action recognition process calls on mental representations that were encoded in an angle-dependent or an angle-invariant manner. In point-light studies, recognition of dynamic biological motion patterns has been found to be impeded by a change in viewing angle^34–36^, suggesting angle-dependent representations. Similarly, in a series of experiments using photographic stimuli, Daems and Verfaillie^37^ found that seeing the same action or pose from a different viewing angle did not reliably facilitate later identification. Body posture representations appear to be angle-specific, such that priming effects dropped halfway when the angle difference between prime and target stimulus was 15°, and almost disappeared with a 30° rotation^37^, with the most finely tuned representations best supporting successful identification of similar postures from similar viewing angles^38^.

Action recognition is also angle-dependent in the visual periphery, but in the opposite direction to previously reported domains. Fademrecht et al.^39^ found that recognition of actions performed by avatars in the far visual periphery was faster at side views than at frontal views, with no difference between front and side in the central visual field. The authors argue that this is because the amount of visible motion information is greater at side views: for example, a punching action seen from the front is just a hand getting larger in front of a body, whereas from the side there is an entire arm stretching out against a contrasting background. Assuming the authors’ hypothesis that more visual information is available when seeing actions from a side view, this visual information would be most helpful in the far periphery, where resolution is low, potentially outweighing the usual frontal advantage. This is particularly relevant to signed language processing, where an interlocutor’s hands are often viewed at lower visual acuity in the visual periphery, due to gaze fixating on the face^40^. Peripheral vision is likely to be engaged more frequently when viewing signers in dialogue from a side angle, for two reasons. Firstly, because the perceived distance between the face and the hands in neutral signing space is greater from a side angle than face-on. Following Fademrecht et al.^39^, signs produced away from the body may not be difficult to understand from the side because more motion information is visible, despite being further into the periphery than when seen frontally. Secondly, picking up backchannels and turn-taking cues in the periphery helps viewers know when to switch gaze when watching two signers in dialogue. Angle dependence has also been shown in the visual recognition of social interactions involving two avatars, like hugging or shaking hands^41^, with each tested action having a clear preferred angle. This is relevant since viewing two or more people interacting in signed language involves a variety of viewing angles, and sign representations encoded in these contexts may also be angle-dependent.

Overall, most results from action/pose classification experiments fit with findings from the subordinate-level object recognition literature^42^. Hamm and McMullen^43^ suggest that complex tasks like semantic classification involve angle-dependent representations, whereas simple identification tasks may call on angle-invariant representations. On the one hand, we might expect sign comprehension to pattern like other complex tasks, since it involves not just visual processing of biological motion, but also linguistic processing (phonological and semantic) to interpret meaning. Thus, we might predict that signed language comprehension recruits angle-dependent representations needed for complex processing. Alternatively, motions with linguistic meaning, like signs and gestures, might bypass this type of complex representation and instead be easier to process than visual actions, thanks to predictability within the linguistic system. This hypothesis could be compared to findings in clinical linguistics which suggest that linguistic narratives are often easier or no harder to process than non-linguistic visual narrative stimuli (e.g. in comics or picture books^44^). This goes against the widespread but mistaken ‘Visual Ease Assumption’, which assumes that non-linguistic visual narrative stimuli elicit fewer processing demands than linguistic narratives. Instead, signed language processing may be less difficult than other action/pose identification tasks precisely because it is linguistic, and therefore pattern like simple recognition tasks.

### Viewing angle in gesture and sign perception

Although signed language input from side angles is encountered in a variety of everyday situations, the role of viewing angle in comprehension is understudied. One exception is Corina et al.^45^, who used a repetition-priming paradigm to examine whether American Sign Language (ASL) representations are angle-dependent or angle-invariant. Deaf signers (early and late) plus hearing non-signers had to decide whether video stimuli depicted an ASL sign or grooming gesture (e.g. head scratching). The authors suggested that deaf participants might show perceptual invariance, i.e. similar priming effects for both same-angle and different-angle prime-target pairs, supposing they would be accessing language-specific representations during the task. However, Corina and colleagues found robust priming effects of angle in both signers and non-signers, i.e. greater priming for same-angle prime-target pairs than different-angle pairs. While deaf signers were slightly more accurate at discriminating signs and actions than hearing non-signers, both groups were affected similarly by the angle variation between prime and target. Thus, Corina and colleagues concluded that the initial stages of sign and gesture recognition do not differ as a function of signed language experience. However, one possible further explanation of deaf signers being affected by angle change is simply that a different side angle presents an adverse condition during sign perception. Alternatively, since group differences were minimal, regardless of viewing angle, it is possible that the gesture/sign discrimination task led to the automatic activation of more complex representations for deaf signers. This may have provided irrelevant phonological information, that in turn weakened group differences, particularly in terms of reaction time.

In terms of other viewing angles of signing, Emmorey and colleagues^46^ found that ASL signers were poor at recognising their own production of signs when self-monitoring. Egocentric or over-the-shoulder viewing angles of signing are encountered relatively rarely, and this result may suggest that signers prefer the most frequent allocentric view of other people’s signing, representing signs accordingly in the mental lexicon. This is a potential difference to action representations, since most actions do not have a clear ‘canonical’ viewing angle, whereas in signing the front angle is the most frequently seen. Lastly, Quinto-Pozos et al.^47^ showed children videos of signers from a 45° over-the-shoulder view in the ASL Perspective Taking Comprehension Test^48^. The authors found that viewing a signer from this perspective was easier than from a regular frontal viewing angle, since no 180° transformation of their signing space is required.

### Mental rotation in sign comprehension

Assuming that mental representations of signs are indeed most likely stored from an allocentric or egocentric view, and not from side views, it is possible that a visuospatial transformation, such as mental rotation, must be performed to successfully map side views onto an internal representation. Several studies have shown mental rotation ability is improved through signed language use [e.g.^49–51^], which is primarily attributed to signers needing to rotate their interlocutor’s spatial descriptions by 180° to understand them. However, see also Secora and Emmorey^52^, who find visuospatial perspective-taking ability (which involves aligning an imagined self-projection in space to the visuospatial perspective of another^53,54^) is more predictive of linguistic perspective-taking than mental rotation ability. Deaf signers do not show the usual mental rotation processing costs in terms of accuracy in the linguistic domain, possibly because the benefits of processing from a frontal viewing angle outweigh the difficulty of mental rotation^51^. Despite this, Brozdowski et al.^55^ show that the 180° transformation required to process classifier constructions in an interlocutor’s signing space when face-to-face is not automatic, takes time, and is cognitively demanding for deaf signers. They also found that interpreting an interlocutor’s signing space was faster from a 90° side angle than face-on, presumably because the smaller rotation takes less time. Brozdowski and colleagues suggest studying sign processing from various angles and looking for a gradual change in performance, under the assumption that this would provide evidence of intermediate analogue representations during processing (see also^56^). Such research could also help answer the question of whether representations of signs are stored from an egocentric (self) or allocentric (other’s) perspective. It remains unclear whether rotation is only required to process explicitly ‘spatial’ uses of signing space, such as indexing referents with a topographic or syntactic function, or if all signs (including, for example, body-anchored signs) need to be rotated to an egocentric perspective to match a representation. If mental rotation is also required when processing signs from non-frontal views in everyday settings such as conversation circles, this usage could also be partly responsible for the mental rotation advantages seen in signers.

The aforementioned rotation advantages are likely due to long-term signed language experience rather than deafness itself, as deaf non-signers do not show advantages in visuospatial cognition^57^, whereas hearing signers do^50^. Age of signed language acquisition is irrelevant to mental rotation ability in both hearing and deaf populations^58^, suggesting rotation is improved through signing experience, even when this starts after infancy. Furthermore, fluent hearing L2 signers have been shown to have better visuospatial working memory compared to non-signing adults^59^, which may include mental rotation. Kubicek and Quandt^60^ found a positive correlation between signed language comprehension and mental rotation abilities, showing that improved rotation already emerges at intermediate levels of sign language proficiency. When looking at less fluent hearing L2 signed language learners, van Loon^61^ found only a limited advantage for intermediate signed L2 learners over spoken L2 learners on a short-term memory task requiring rotation. Likewise, beginner L2 signed language learners showed no improvement in mental rotation at the end of one ASL course^62^. If mental rotation is involved in sign comprehension from side viewing angles, the evidence suggests that L2 learners need to reach a certain level of fluency before their mental rotation ability improves enough to in turn feed back into their ability to rotate during signed language comprehension.

### Present study

Building on the work of Corina et al.^45^, we investigate angle specificity during semantic processing of signed language, i.e. a more challenging task than sign/gesture discrimination. This may shed light on the degree to which linguistic representations differ from action representations. While action recognition is a complex visual task, semantic processing must draw on further resources still to decode phonological input and, in signed language processing, attend to multiple articulators simultaneously. The present study addresses the role of viewing angle in both L1 and L2 signed language processing, using a semantic categorisation task to ensure linguistic processing, within a distance priming paradigm, meaning prime and target trials were separated by many intervening trials. We chose distance priming for two reasons. Firstly, we wanted to expand on the work of Corina and colleagues, who used repetition priming, by employing a different priming paradigm. Secondly, we believe distance priming is a more ecologically valid method than repetition priming, as distance priming more accurately reflects how the same signs may recur within signed language conversation (i.e. with a number of other intervening signs), rather than consecutive repetition of individual signs. We make use of stimuli filmed from five different viewing angles and a range of prime-target combinations (same target angle, easier target angle, harder target angle), to detect any incremental effects of angle.

Firstly, we aim to find out whether non-frontal viewing angles are an adverse condition for semantic processing of signs. Secondly, if side angles do impede sign comprehension, we ask whether hearing L2 learners are disproportionately affected. Lastly, we ask what mechanism is involved in successfully comprehending signs presented from side angles. If the results show similar priming across all three conditions of prime-target combinations, we would interpret this as evidence of rich three-dimensional representations of signs from all viewing angles. If priming is greatest when the same angle is seen again at target, this would be evidence for angle-dependent representations, similar to action recognition (as described above). Alternatively, if the most difficult condition is when the sign is seen again from a new ‘harder’ side angle, this would suggest that recognition of signs from side angles requires a mental transformation.

## Methods

### Participants

Forty-five right-handed British Sign Language (BSL) signers participated in the experiment (31 women, 13 men, 1 non-binary; average age 32; range 19–54; see Table 1). To distinguish the potential roles of deafness, language background and degrees of L2 fluency in processing signs from visual angles, participants belonged to one of four groups: L1 deaf signers (exposed to BSL from birth, *n* = 12), L1 hearing signers (exposed to BSL from birth,* n* = 9), advanced hearing L2 signers (minimum BSL Level 6 or equivalent, *n* = 11), and intermediate hearing L2 signed language learners (minimum BSL Level 2 or equivalent, *n* = 13). Signers were recruited from the deaf/BSL learning communities around Birmingham, London and Edinburgh via our participant database, outreach at a local deaf festival (Deaffest) and social media adverts. Informed consent was obtained from all participants, who were reimbursed £12/hour for taking part.

**Table 1:** Overview of participant groups, demographics, and language backgrounds.

Group	*N*	Mean age	Mean age at BSL 1st exposure	Mean BSL use duration	Mean BSL self-rating (1–7)	Mean BSL-SRT score (0–44)
L1 Deaf	12	28;7 (8;9)	0;0 (0;0)	28;7 (9;0)	6.3 (0.9)	28.2 (3.1)
L1 Hearing	9	30;6 (9;4)	0;0 (0;0)	30;6 (6;10)	5.4 (0.7)	20.0 (3.0)
L2 Advanced	11	37;3 (8;6)	24;2 (9;0)	12;6 (6;10)	5.4 (0.9)	19.3 (2.2)
L2 Intermediate	13	33;2 (6;5)	23;9 (4;8)	8;8 (5;8)	3.4 (1.2)	12.0 (5.5)

Mean ages in years; months, standard deviation in brackets. The L2 Intermediate group self-rated their BSL proficiency significantly lower than the other three groups. The BSL Sentence Reproduction Test significantly distinguished all groups from each other, apart from the L1 Hearing and L2 Advanced groups.

### Item selection

240 BSL signs were selected from a range of sources, including beginners’ resources aimed at adult L2 signed language learners^63,64^; the BSL Communicative Development Inventories^65^; and those signs for which norms were available: 69% of items had iconicity ratings; 28% had familiarity and age-of-acquisition norms^66,67^. High-familiarity and early age-of-acquisition signs were prioritised, to increase the likelihood that intermediate L2 learners would be familiar with all items presented. All signs were nouns and included mouthing (nouns are commonly mouthed in BSL^68^).

### Stimuli creation

**Figure 1:**
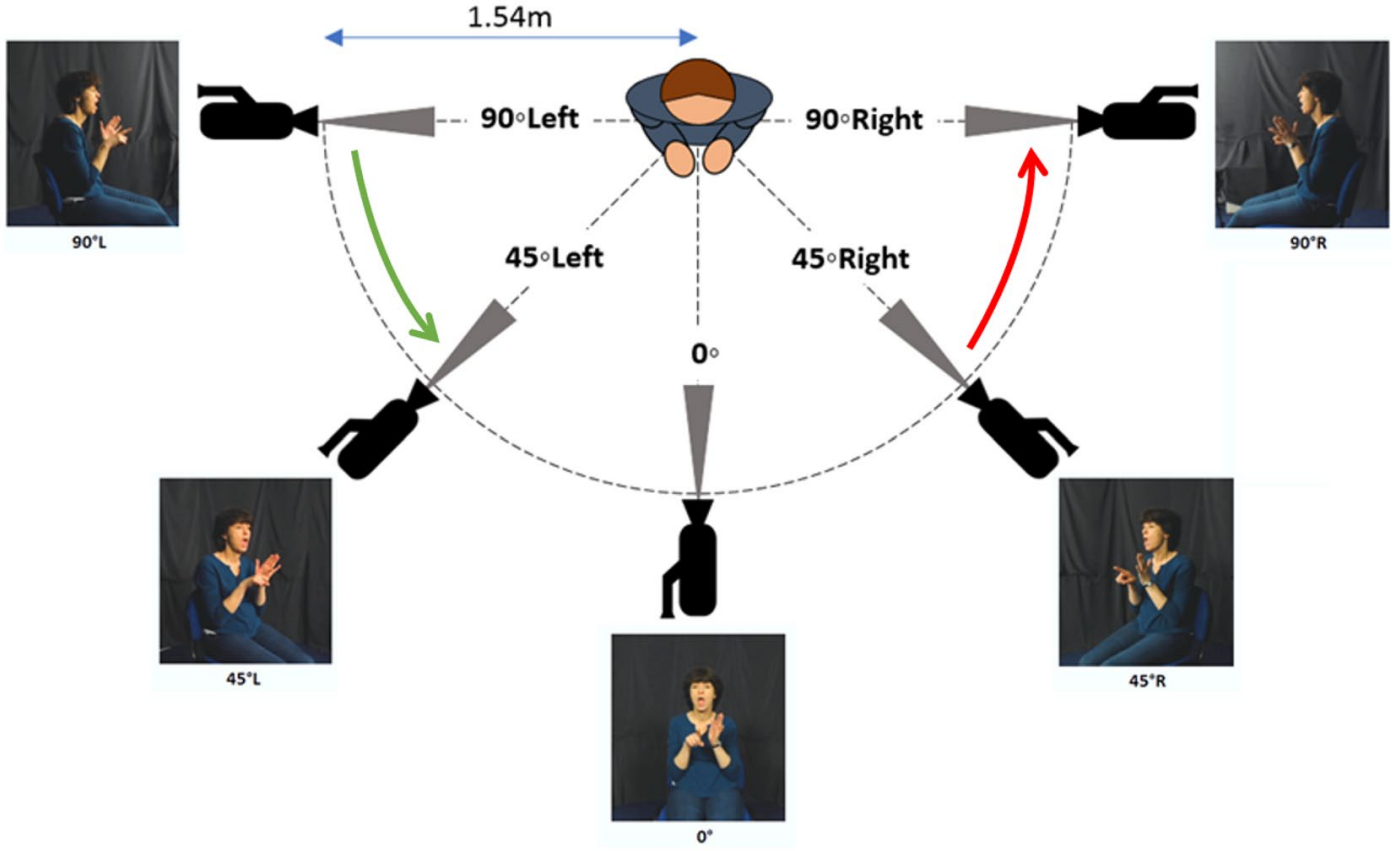
Positioning of the five cameras relative to the sign model. Example stimulus ALARM shown at each angle. Green arrow: example prime-target pair where the target angle (45°L) is ‘easier-by-45°’ than the prime angle (90°L). Red arrow: example prime-target pair where the target angle (90°R) is ‘harder-by-45°’ than the prime angle (45°R).

A total of 1200 BSL video stimuli were created (240 from each angle) using five Logitech C920 HD Pro cameras. Cameras were arranged in a 180° arc, with each one situated 1.2 m from the ground and equidistant (1.5 m) from the sign model (Fig. 1; using the nose as the central fixation point). The sign model was lit by a single box light placed behind the frontal camera; we allowed lighting conditions to vary naturally with the angles. Time-locked simultaneous filming was achieved via Python code^69^ which ensured that a single production of each sign was recorded from all 5 camera angles simultaneously. The sign model was a right-handed deaf L1 BSL signer with deaf signing parents. Stimuli were recorded individually in randomised order. Final Cut Pro X was used to adjust contrast, brightness, and to colour correct the videos, as well as to crop all videos to 382 × 400 pixels so that the signer was centrally positioned. Videos were clipped further such that the first visible frame showed the sign model’s hands begin to move from the lap, and the final frame was when the hands returned to the lap, since reaction time is to be measured from stimulus onset.

### Design

**Figure 2:**
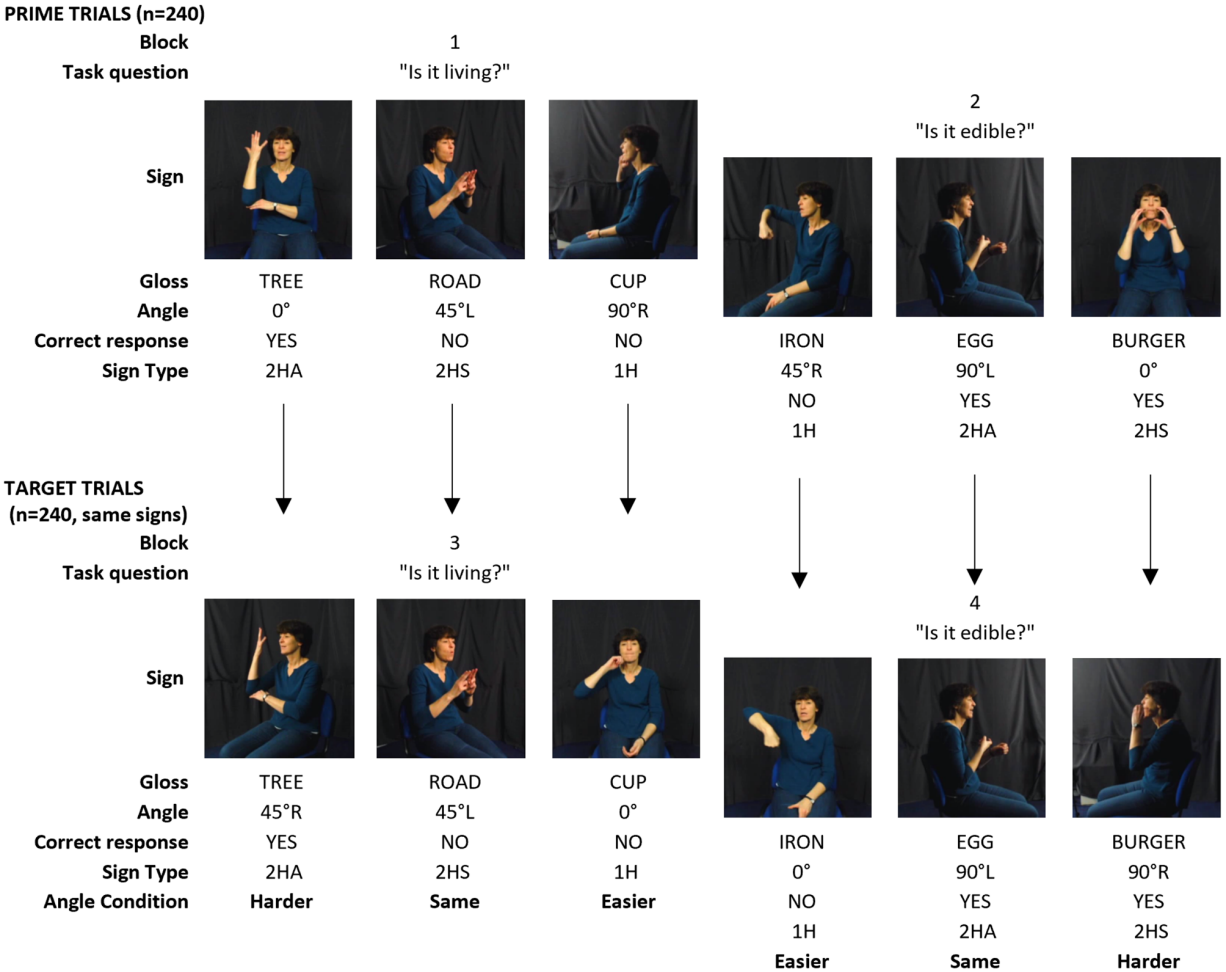
Experiment design showing three example stimuli per block. The top row shows six prime trials seen in the first half of the experiment; the bottom row shows the corresponding target trials seen in the second half of the experiment.

This long-distance priming study consisted of 480 total trials with 240 unique sign items viewed twice in the experiment: once in the first half as a prime (in block 1 or 2), and again in the second half as a target (in block 3 or 4). This means that each prime trial and its target were always separated by an intervening block of trials (see Fig. 2). Trial order within each block was randomised for each subject. This means that e.g. trial 1 in block 1 could be a prime for e.g. target trial 310 in block 3, and trial 2 in block 1 could be a prime for e.g. target trial 285 in block 3. Blocks alternated between two semantic decision tasks: ‘is it living?’ and ‘is it edible?’ The first half of the experiment (blocks 1 and 2) provides a baseline no-prime condition.

The primary experimental manipulation was the degree of change in viewing angle between the prime trial and the target trial, henceforth ‘prime-target disparity’. Prime-target pairs for each item were counterbalanced across three categories of prime-target disparity: *n* = 80 pairs with no/0° change (control condition); *n* = 80 pairs with a 45° change between prime and target; and *n* = 80 pairs with a 90° change between prime and target. Three lists were created such that each item appeared in each of the three prime-target disparity conditions (no/0°, 45° and 90° change). All participants were exposed to all signs; the only variation was the angle from which the signs were presented.

Pairs with a 45° or a 90° prime-target disparity were also counterbalanced such that the order was reversed 50% of the time across versions. Assuming that comprehension is easiest at the front viewing angle, more difficult at 45° and hardest at 90°, we also hypothesised that a relevant factor might be the directionality of prime-target-pairs, i.e. whether the target stimulus was seen from a ‘harder’ angle; an ‘easier’ angle; or the ‘same’ angle as the prime stimulus. Items were balanced across blocks to ensure equivalent proportions of three sign types, i.e. how the hands are configured in terms of symmetry during signing. Thus the three basic sign types are one-handed (1H), two-handed symmetrical (2HS) or two-handed asymmetrical (2HA; see Fig. 2 for examples of each)^70^. We considered this particularly important because different sign types may be easier or harder to process depending on the visual angle (e.g. for 2HS and 2HA signs presented from 90° angles, one hand could partially occlude the other, reducing phonological input).

### Procedure

Participants received instructions in both BSL and written English and then completed a practice block of 10 trials. Each trial was preceded by a central fixation cross for 400 ms, with reaction times (RTs) measured from video onset. Participants could take a self-paced break every 30 trials if desired. At the end of each block, participants were reminded of the change in the semantic decision for the upcoming block. Responses were made via keyboard button presses, with the location of the match/no match keys (F/J) counterbalanced across participants. Stimuli were presented using E-Prime 2.0^71^.

After completing the priming study, participants performed a short mental rotation task, in which they were asked to judge whether two shapes were identical or mirror images, by mentally rotating them around the vertical axis (the same vertical direction of rotation needed in sign processing). Participants saw 48 pairs of shapes from Ganis and Kievit^72^, presented in E-Prime 2.0, and responded via button press (‘identical or mirror image?’). Accuracy and reaction time were measured.

Participants then completed the BSL Sentence Reproduction Test^73^ as a measure of BSL fluency. The test features 44 BSL sentences of increasing difficulty which are shown to the participant as video clips and must be reproduced as accurately as possible. Each sentence is marked as either ‘correct’ if it is reproduced exactly as presented in full, or ‘incorrect’ if there is a mistake anywhere in the sentence, with limited acceptable deviations. The test is a combined measure of receptive and productive skills and has been shown to successfully distinguish L1 and L2 signers.

Participants also completed a pre-experiment language background questionnaire and a post-experiment questionnaire. The latter aimed to collect more detail about how participants acquired BSL, and their past and present BSL use in different social contexts (e.g. alone/face-to-face/in groups), as a proxy estimate of exposure to signs from different visual angles. Importantly, when first learning BSL, hearing L2 signers reported considerably less exposure to situations with side-on viewing angles (e.g. passively watching dialogues) compared to situations with face-to-face signing. L1 signers, on the other hand, reported only slightly reduced early exposure to side views of BSL compared to face-on (Table 2; for full results, see supplementary OSF repository).

**Table 2:** Mean self-ratings of frequency of exposure to types of BSL input, standard deviation in brackets (0 = ‘Never’; 1 = ‘Rarely’; 2 = ‘Occasionally’; 3 = ‘Often’; 4 = ‘Frequently’).

BSL input type	When first learning BSL		Current use of BSL	
	L1	Hearing L2	L1	Hearing L2
One signer, face-to-face (0°)	3.70 (0.65)	2.48 (1.09)	3.55 (0.50)	2.88 (1.30)
Two signers, side-on (~ 60–90°)	3.45 (1.16)	1.85 (1.20)	3.30 (0.83)	2.71 (1.24)

### Ethics, transparency and openness

This study was reviewed and approved by University of Birmingham Science, Technology, Engineering and Mathematics Ethical Review Committee (ERN_18-1170) and performed in accordance with the Declaration of Helsinki. All participants provided their written informed consent to participate in this study and written informed consent was sought from our sign model to use images in this publication. We report all data exclusions, all manipulations, and all measures in the study, and we attempt to follow JARS^74^. This study’s design and its analysis were not pre-registered. In line with standards of reproducible research, the scripts and data are made available with this publication and can be retrieved on the following publicly accessible repository: https://osf.io/njmhb. We used R version 4.0.5^75^ and the packages lme4 version 1.1.27.1^76^ and afex version 0.28.1^77^ for the linear mixed effects model analysis reported below. For data processing we used the package tidyverse version 1.3.1^78^.

### Statistical analysis

We excluded thirteen items for low accuracy (< 70%; 5.4% of 240 items), which were predominantly low-frequency signs or regional variants, and 22 trials for extreme RTs (< 500 ms or > 5000 ms; 0.1% of total trials). For the RT analysis, incorrect responses were excluded (*N* = 768 trials; 7.5%). For the prime-target disparity analysis, we also excluded the target pair of any prime trials which were already excluded due to extreme RTs/being incorrect. To analyse our data, we used linear mixed effects models because the distance priming paradigm is a repeated measures design where we have multiple non-independent data points per participant, per angle condition, and per item.

First, to investigate whether non-frontal angles are an adverse condition for signed language comprehension, we analysed prime trials only. For the accuracy analysis, we used mixed logistic regression because our response variable was binary (correct, incorrect). The fixed effects predictors were Angle (three levels: 0°, 45°, 90°; treated as a categorical variable), Group (four levels: L1 Deaf, L1 Hearing, L2 Advanced, L2 Intermediate), and BSL-SRT score (numeric), as a more granular measure of fluency. Angle was Helmert-coded since it is an ordinal factor, while Group was treatment-coded with L1 Deaf as the reference level. To assess whether the less fluent signers were disproportionately affected by non-frontal angles, we also considered interactions of Angle * Group, plus Angle * BSL-SRT. The random effects component of our mixed models included random intercepts for Participants and Items^79^, as well as by-Participant and by-Item random slopes for the critical within-subjects variable Angle. We log-transformed reaction times to better fit the normality assumptions of regression models. The model structure was largely the same as for the Accuracy analysis. Because responses to ‘yes’ trials were overall faster (*M* = 1873 ms, SD = 473.4) than ‘no’ trials (*M* = 2031 ms, SD = 515.1), we included this as an additional factor.

To investigate whether comprehension of signs seen from side angles is achieved via mental rotation, we compared responses to the prime trial with the target trial for each sign. To do this, we calculated the change in accuracy between prime and target by subtracting PrimeAccuracy-TargetAccuracy, and the amount of priming by subtracting PrimeLogRT-TargetLogRT, for each sign per participant. Since we predict that comprehension is easiest at 0° and hardest at 90°, we hypothesise that the angle relationship between prime-target pairs will be important. Therefore, we fitted models with the predictor Difficulty (Easier angle, Same angle, Harder angle) of prime-target disparity in viewing angle, to assess the priming effect on accuracy and speed. Difficulty was also Helmert-coded as an ordinal factor. Again, as a more granular measure, we also ran models with a five-level predictor that takes into account both the direction *and* the size of the change in angle between prime and target (easier-by-90°, easier-by-45°, same-0°, harder-by-45°, harder-by-90°). Otherwise, the model structure remained the same as for analysis of the prime data.

For each fixed effect, we report *p*-values based on likelihood ratio tests of the full model with the fixed effect in question against a comparable null model without the fixed effect in question. All planned contrast comparisons were Bonferroni-corrected to avoid an inflated Type I error rate.

## Results

First, we analysed the prime data from the first half of the experiment alone, starting with the accuracy analysis, to get a baseline.

### Prime trials: accuracy

**Figure 3:**
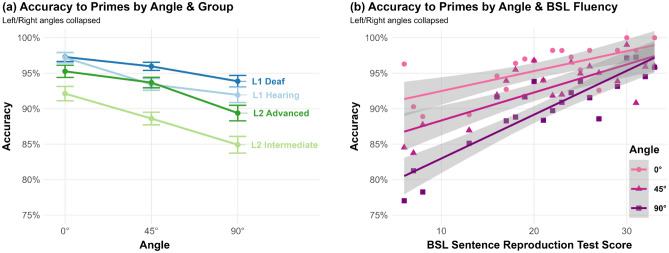
Accuracy on Prime trials by Angle and (**a**) Group; (**b**) BSL-SRT Score. Standard error 95% confidence intervals plotted.

The average accuracy across all participants was 92.2% (SD = 4.6%, range: 80.2–98.3%). Angle was a statistically significant predictor of Accuracy (χ^2^(1) = 17.20, *p* < 0.001), with all groups most accurate to signs presented from 0° (*M* = 95.3%, SD = 4.1%), less accurate at 45° (*M* = 92.8%, SD = 4.99%) and worst at 90° (*M* = 89.8%, SD = 5.92%; Fig. 3a). Pairwise comparisons using *Z*-tests, corrected with the Bonferroni method, indicated that performance at 90° was significantly less accurate than at both 0° (*Z* = 4.53, *p* < 0.001) and at 45° (*Z* = 4.2, *p* = 0.001), though 0° and 45° did not differ significantly (*Z* = 1.28, *p* = 0.6). Group was also a statistically significant predictor of Accuracy (χ^2^(3) = 15.36, *p* = 0.002), with pairwise comparison tests indicating that the L2 Intermediate group (*M* = 88.3%, SD = 5.15%) was significantly less accurate than all other groups (vs. L1 Deaf: *M* = 95.6%, SD = 1.68%, *Z* = 5.43, *p* < 0.001; vs. L1 Hearing: *M* = 93.9%, SD = 3.52%, *Z* = 4.03, *p* = 0.0003; vs. L2 Advanced: *M* = 92.7%, SD = 3.25%, *Z* = 2.86, *p* = 0.0255), though there were no other group differences. The interaction between Angle and Group was not significant (χ^2^(3) = 4.49, *p* = 0.21), which suggests that there were no marked differences in how the different signing groups performed with respect to the angle conditions, i.e., all were about equally affected. Repeating the model using BSL-SRT score as a more fine-grained predictor of BSL fluency than Group, there was a significant interaction between Angle and BSL-SRT score (χ^2^(2) = 8.58, *p* = 0.014), suggesting that the accuracies for less fluent signers were disproportionately lower at non-frontal angles (Fig. 3b). We also fit a model with Angle as a five-level factor instead of three-level, to account for potential effects of viewing from the Right or the Left side. However, there were no significant pairwise differences between the 45°R and 45°L angles (*M* = 92.1%, SD = 5.7%, vs M = 93.4%, SD = 5.44%, *Z* = 0.8, *p* = 1), nor between the 90°R and 90°L angles (*M* = 90.2%, SD = 7.26%, vs. M = 89.5%, SD = 6.39%, *Z* = 0.44, *p* = 1).

### Prime trials: reaction time

**Figure 4:**
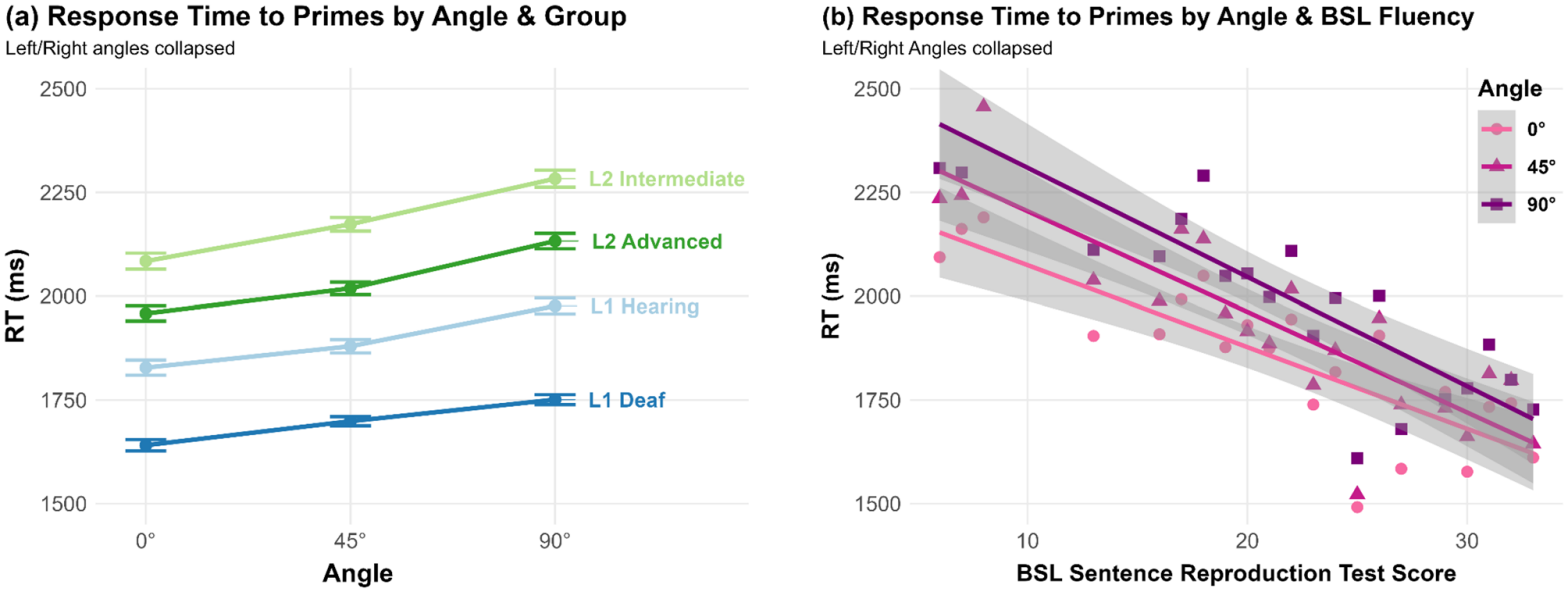
Response Time to Prime trials by Angle and (**a**) Group; (**b**) BSL Sentence Reproduction Test Score. Standard error 95% confidence intervals plotted.

Angle was also a statistically significant predictor of LogRT (χ^2^(2) = 14.92, *p* < 0.001), with all groups slower to respond to signs presented further from 0° (0°: 1884 ms, SD = 222 ms; 45°: 1952 ms, SD = 247 ms; 90°: 2046 ms, SD = 268 ms). Pairwise comparisons indicated that response time significantly differed among all angles (0° vs. 45°: *Z* = 2.42, *p* = 0.047; 0° vs. 90°: *Z* = 6.85, *p* < 0.001; 45° vs 90°: *Z* = 5.83, *p* < 0.001). Group also significantly predicted LogRT (χ^2^(3) = 46.86, *p* < 0.001), with L1 Deaf signers (*M* = 1701 ms, SD = 118 ms) significantly faster than all other groups in pairwise tests (vs. L1 Hearing, *M* = 1895 ms, SD = 164 ms, *Z* = 2.96, *p* = 0.018; vs. L2 Advanced, *M* = 2040 ms, SD = 137 ms, *Z* = 6.1, *p* < 0.001; vs. L2 Intermediate, *M* = 2190 ms, SD = 194 ms; *Z* = 8.84, *p* < 0.001). The L2 intermediate group was also significantly slower than the L1 Hearing group (*Z* = 4.9, *p* < 0.001), but not the L2 Advanced group (*Z* = 2.46, *p* = 0.084). There was a significant main effect of Response (χ^2^(1) = 16.79, *p* < 0.001), with Yes responses (*M* = 1873 ms, SD = 473 ms) faster than No responses (*M* = 2031 ms, SD = 515 ms). The interaction between Angle and Group was not significant (χ^2^(6) = 7.95, *p* = 0.24; Fig. 4a), suggesting that response times for less fluent signers were not disproportionately lower at non-frontal angles (Fig. 4a). Results were similar when repeating the model with BSL-SRT score as a more fine-grained predictor of BSL fluency than Group: BSL-SRT score was a significant predictor of response speed (χ^2^(1) = 28.72, *p* < 0.001), but the interaction between Angle and BSL-SRT score was not significant (χ^2^(2) = 5.29, *p* = 0.07; Fig. 4b). Again, we fit a model with Angle as a five-level factor instead of three-level, to account for potential effects of viewing from Right or the Left. However, there were no significant pairwise differences between the 45°R and 45°L angles (*M* = 1969 ms, SD = 503 ms, vs *M* = 1921 ms, SD = 508 ms; *Z* = 1.04, *p* = 1), nor between the 90°R and 90°L angles (*M* = 2039 ms, SD = 534 ms, vs. *M* = 2031 ms, SD = 505 ms; *Z* = 0.34, *p* = 1).

### Target trials: accuracy improvement

**Figure 5:**
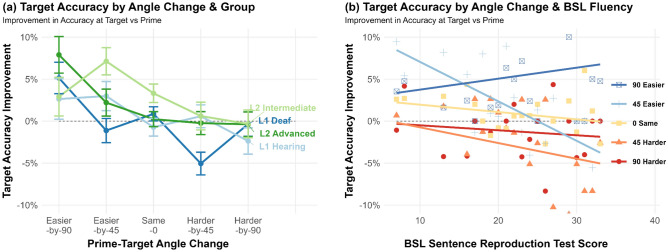
Target accuracy improvement by the Angle Change between prime and target trials and by (**a**) Group; (**b**) BSL-SRT Score. Standard error 95% confidence intervals plotted.

Difficulty of the target trial (Same angle, Easier angle, Harder angle) was a significant predictor of change in accuracy between prime and target (χ^2^(2) = 9.22, *p* = 0.01), with the highest target accuracy improvement for signs shown from an Easier angle (3.5% increase; Same angle: 1.1% increase; Harder angle: -1% decrease). Pairwise comparisons indicated that the improvement in the Easier condition was significantly greater than the other two conditions (vs. Same: *Z* = 3.09, *p* = 0.006; vs. Harder: *Z* = 4.84, *p* < 0.001), while the Same and Harder conditions did not differ from each other (*Z* = 2.23, *p* = 0.078). Group was also a significant predictor of improved accuracy at target (χ^2^(3) = 15.47, *p* = 0.001), with greater accuracy gains at target for less fluent groups. Pairwise comparisons revealed that the L2 intermediate group improved significantly more than the L1 Deaf group (*Z* = 3.94, *p* < 0.001). The interaction between Difficulty and Group was not significant (χ^2^(6) = 6.39, *p* = 0.38), suggesting that there were no clear differences across the four groups in how the difficulty of the target angle relative to the prime angle affected target accuracy, i.e. all groups improved at similar levels across conditions. However, when repeating the model with the more gradient predictor ‘Angle Change’, which also considers the size of the angle change, there was a stronger main effect of Angle Change than of Difficulty alone (χ^2^(4) = 25.34, *p* < 0.001), whereby easier-by-90° changes saw the most improvement (+ 4.7%), and the two harder angle changes a slight reduction in accuracy (harder-by-45°: −1.1%; harder-by-90°: −0.8%). Here there was a significant interaction between Angle Change and Group (χ^2^(12) = 25.37, *p* = 0.013), suggesting that improvements at target for different angle changes varied by group (Fig. 5a). Specifically, less fluent signers did not benefit as much from the easier-by-90° Angle Change compared to more fluent signers. This was also the case when using BSL-SRT score as a predictor instead of Group (see Fig. 5b).

### Target trials: RT priming

**Figure 6:**
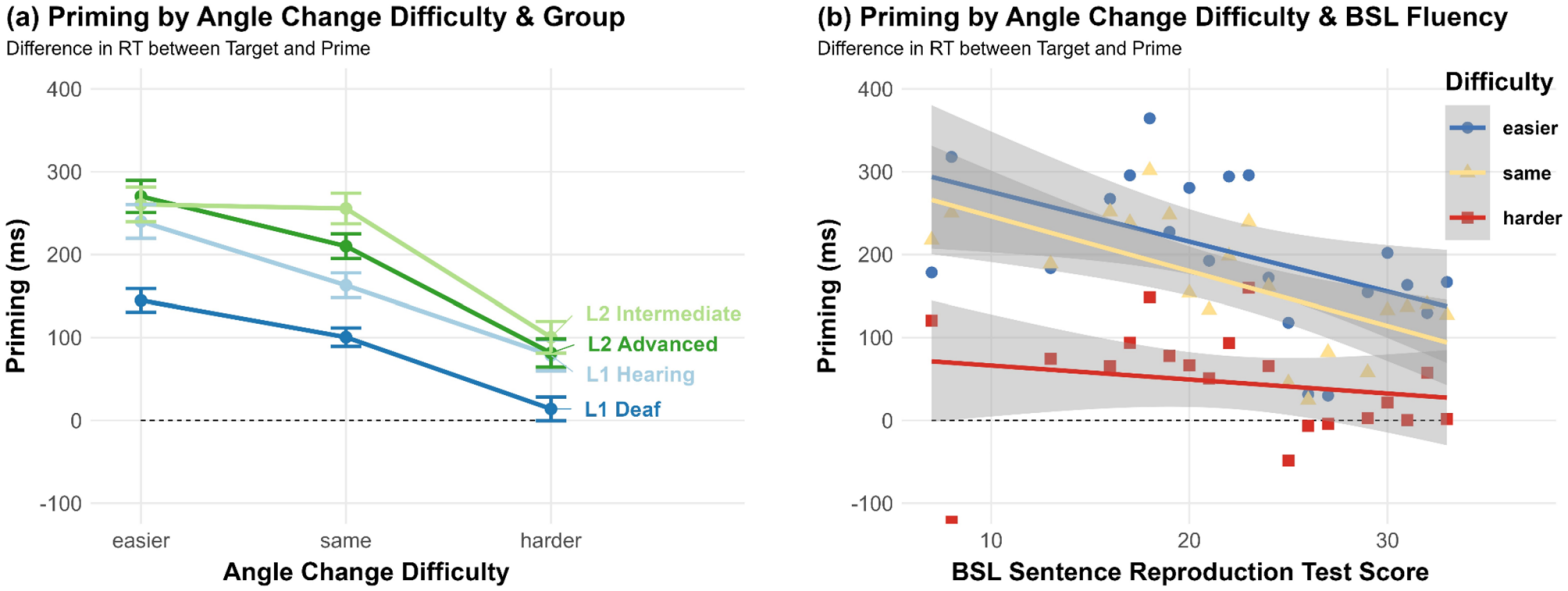
RT Priming by Difficulty condition and (**a**) Group; (**b**) BSL-SRT Score. Standard error 95% confidence intervals plotted.

Difficulty condition also had a significant effect on how much faster target responses were versus prime trials (χ^2^(2) = 159.17, *p* < 0.001), with much greater priming in the Same angle (182 ms) and Easier angle (226 ms) conditions than the Harder angle condition (68 ms; Fig. 6a). Pairwise comparisons indicated that priming in the Harder condition was significantly lower than the other two conditions (vs. Same: *Z* = 11.96, *p* < 0.001; vs. Easier: *Z* = 10.8, *p* < 0.001), while the Same and Easier conditions did not differ from each other (*Z* = 0.86, *p* = 1). There was also a significant main effect of Group (χ^2^(3) = 8.93, *p* = 0.030), with an inverse relationship between fluency and amount of priming, i.e. less fluent signers get greater benefit from seeing signs a second time. Pairwise comparisons revealed that the L2 intermediate group (207 ms) showed significantly more priming than the L1 Deaf group (86 ms; *Z* = 2.92, *p* = 0.021). The interaction between Difficulty and Group was not significant (χ^2^(6) = 5.02, *p* = 0.542), nor was the interaction between Difficulty and BSL-SRT Score (χ^2^(2) = 3.82, *p* = 0.15; Fig. 6b). When repeating the model with the more gradient predictor ‘Angle Change’, which also considers the size of the angle change, there was a stronger main effect of Angle Change than of Difficulty alone (χ^2^(4) = 185.29, *p* < 0.001), whereby there was only minimal priming in the two harder angle change categories (harder-by-45°: 98 ms; harder-by-90°: 21 ms) compared to the other conditions (same-0°: 182 ms; easier-by-45°: 206 ms; easier-by-90°: 264 ms). There was no significant interaction between Angle Change and Group (χ^2^(12) = 15.89, *p* = 0.196), suggesting that priming at target for different angle changes did not vary by group. Planned comparisons revealed that the L1 Deaf group was able to take considerable advantage of the easier-by-90° condition (*M* = 203 ms), with significantly more priming than when there was no angle change (*M* = 101 ms; *Z* = 3.05, *p* = 0.035), while all other groups could not.

### Sign type

**Figure 7:**
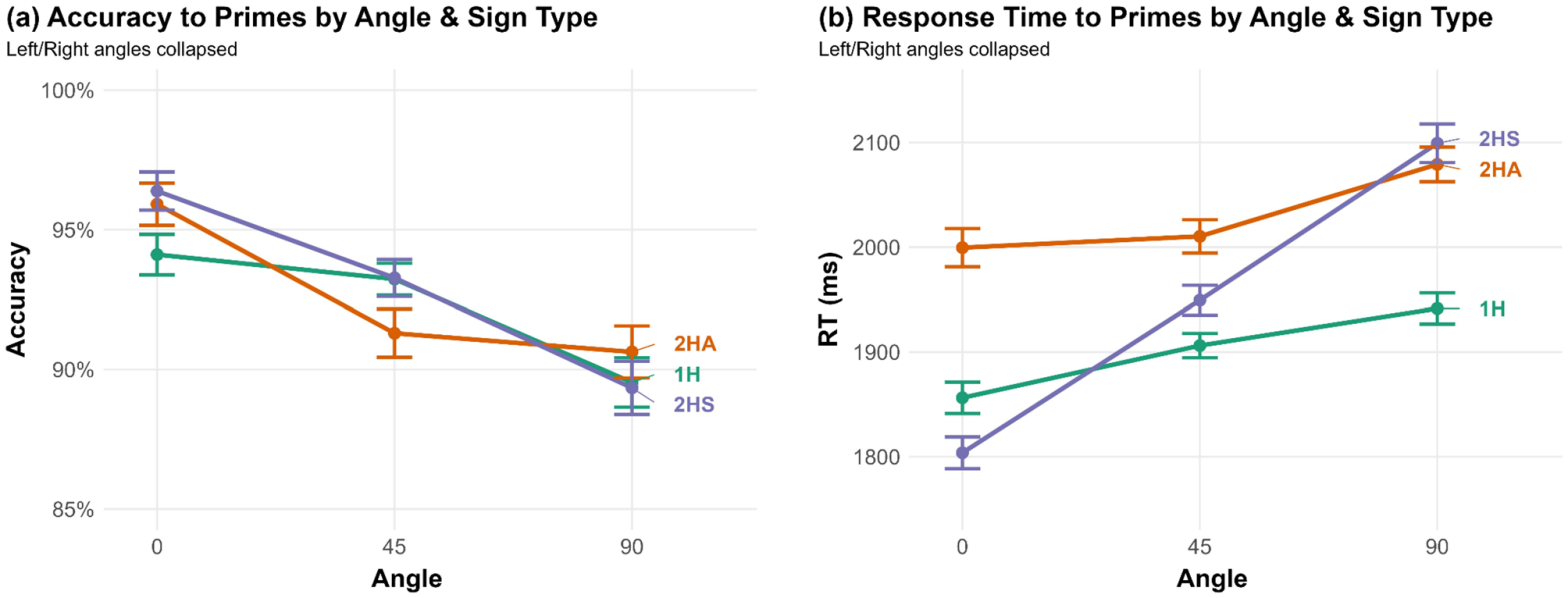
Effect of Angle and Sign Type on (**a**) Accuracy; (**b**) Reaction Time. Standard error 95% confidence intervals plotted.

Experiment items were categorised into one of three Sign Types: one-handed (1H), two-handed symmetrical (2HS) and two-handed asymmetrical (2HA) signs. Since 2HA signs are the most phonologically complex, we expect these to be the most difficult to process. There was no effect of Sign Type on Accuracy to primes (χ^2^(2) = 0.52, *p* = 0.77), and there was no interaction between Sign Type and Angle (χ^2^(4) = 6.87, *p* = 0.143; Fig. 7a). Sign Type had a significant effect on RT to prime trials (χ^2^(2) = 10.33, *p* = 0.006), with faster responses to 1H signs (*M* = 1948 ms) than 2HA signs (*M* = 2081 ms) overall (*Z* = 3.28, *p* = 0.003). However, there was no significant interaction between Sign Type and Angle (χ^2^(4) = 6.91, *p* = 0.141).

In terms of RT (Fig. 7b), despite the lack of significant interaction between Sign Type and Angle, it is notable that 2HS signs show a considerable angle effect, with responses at 90° 295 ms slower than at 0°. In contrast, 1H signs were only 85 ms slower at 90° vs. 0°, and 2HA signs were only 80 ms slower at 90° vs. 0°. Sign Type had no effect on the change in accuracy between prime and target (χ^2^(2) = 4.06, *p* = 0.132), and no effect on RT priming (χ^2^(2) = 2.22, *p* = 0.330).

### Mental rotation

**Figure 8:**
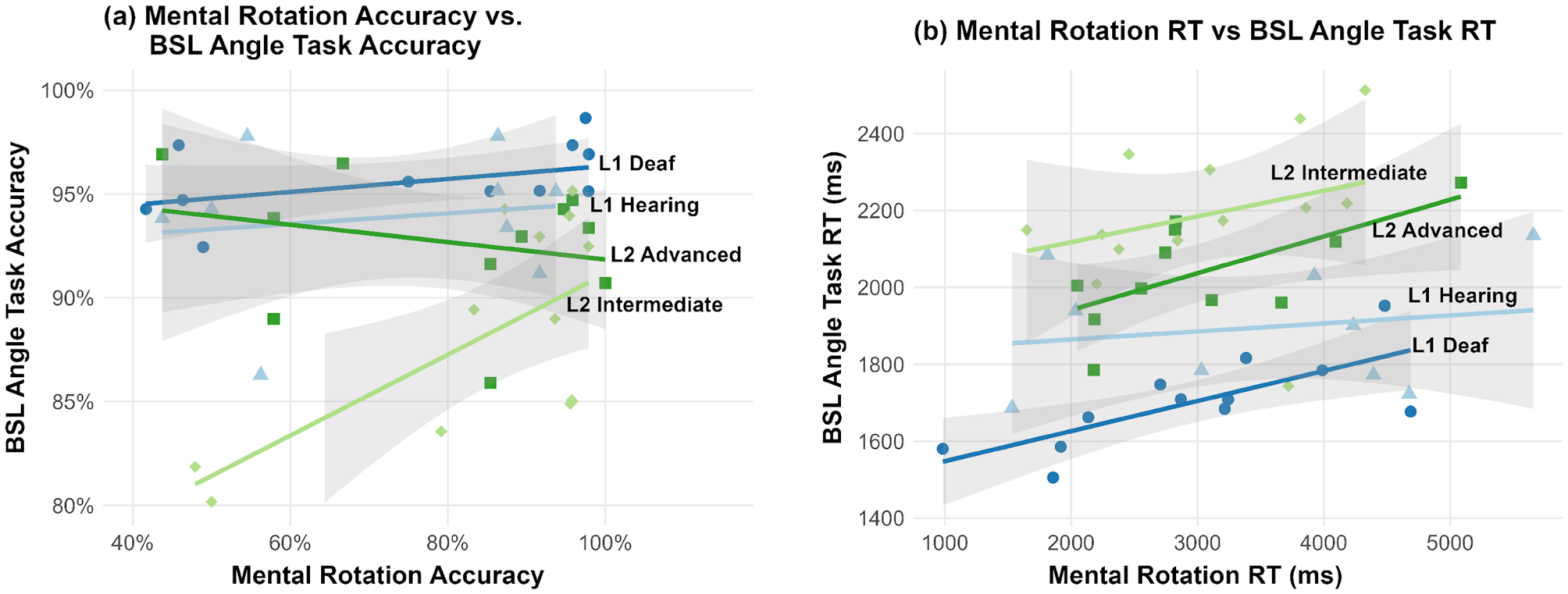
Performance on the BSL Angle task versus Mental Rotation task in terms of (**a**) Accuracy; (**b**) Reaction Time.

Since we hypothesised that mental rotation might be required to process the BSL signs presented from side angles in the semantic decision task, we were also interested in participants’ mental rotation ability on a block rotation task. We included this Mental Rotation score in all previous models as a fixed effect but it was not a significant predictor of accuracy or response time. Many participants found the block rotation task difficult (overall mean accuracy = 79.1%, participant accuracies ranged from 42 to 100%). Here we additionally compare participants’ performance in the first half of the BSL semantic decision task to their performance on the block rotation task. Comparing participants’ mean mental rotation accuracy scores to their mean BSL task accuracy (Fig. 8a), there was a moderate positive Pearson’s correlation for the L2 Intermediate learner group: *r* = 0.624. Correlations for the three BSL-fluent groups are not as strong, in part because they all performed close to ceiling in the BSL task. L1 Deaf signers were the next highest: *r* = 0.427. RTs in the BSL task were positively correlated with RTs in the mental rotation task for all groups (Fig. 8b). The strongest correlation was for L1 Deaf signers: *r* = 0.734, followed by L2 Advanced learners: *r* = 0.65. However, correlations were weaker for L2 Intermediate learners: *r* = 0.332, and L1 Hearing signers: *r* = 0.192. While correlation is not evidence of causation, these results provide some suggestion that poorer performance on the BSL task involving multiple viewing angles may be in part due to poorer mental rotation skill.

## Discussion

### Extending angle-specificity to signed language processing

From the responses to prime trials, we find evidence that side viewing angles are an adverse condition for semantic processing of signs, negatively affecting both L1 and hearing L2 signers. Specifically, results from the first half of the distance priming experiment show that before signs have been primed from a particular viewing angle, processing is fastest and most accurate from the front (0°), with a significant gradient increase in reaction time and decrease in accuracy from 0° to 45° to 90° in turn. Thus, viewing angle matters in signed language processing.

These results suggest that existing representations of signs in the mental lexicon are likely stored from a frontal view (of the interlocutor, not of the self) and that viewing other angles incurs a processing cost. This cost is greater the further the viewing angle deviates from 0°. The angle-dependent linguistic processing seen in the first half of the present study extends the angle specificity results of Corina and colleagues^45^ for sign/gesture discrimination. Importantly, it also extends the broader literature that finds angle specificity in a wide range of recognition domains, including for objects^42^, faces^80^, postures and actions^37^, spatial layouts^81^, and social interactions^41^. Our results, using a semantic decision task, support the idea that angle-dependent effects occur when a task requires more complex processing or involves subordinate categorisation, whereas simpler discrimination and identification tasks or those involving superordinate categorisation are more likely to be angle-invariant^43^.

Further, most research on angle specificity in other domains refers to within-experiment specificity, calling on short-term mental representations of, for example, unique objects or pose stimuli created in the process of doing the experiment task. In contrast, the results from the first half of our experiment inform us about the nature of pre-existing linguistic representations: our BSL signs were already known to the participants. We suggest that sign representations are usually created and stored at a frontal 0° angle, likely because this view is seen first and most frequently thereafter. This is in contrast to everyday object and pose recognition, where the frontal viewing angle is not necessarily the first angle seen. Furthermore, we may see objects across more angles than we do with signs. As mentioned earlier, signers will generally orient themselves to face an interlocutor where possible, however, when viewing objects, a perceiver will rarely reorient themself to a frontal view. A possible analogy here is spoken words being stored with the pronunciation or accent heard when the word is first encountered, with representations changing as a more diverse range of pronunciations is heard (cf. Goldinger)^82^.

### Mechanisms of processing non-frontal linguistic input

In other recognition domains, angle invariance has generally been found for simpler tasks, and angle specificity for more complex tasks. In the present study, in terms of the difference between prime and target trials, a rich, 3D, angle-invariant representation account would have predicted equivalent levels of priming in each difficulty condition. This, however, is not what we found. Further, a purely angle-dependent account of the results predicts that priming would be greatest in the Same angle condition, yet the Easier angle condition showed similar if not greater levels of priming. Therefore, our results are not as simple as a finding of either angle-dependent representations versus angle invariance, like the dichotomy seen in much of the previous orientation specificity literature. However, what we see instead is a language-specific effect. Specifically, we interpret our distance priming results as evidence that existing linguistic representations for BSL signs are stored first and foremost from a frontal view, and whenever a side angle is encountered, it must be mentally rotated to this front angle.

We saw greatly reduced priming in the Harder angle condition (where the prime is an easier, more frontal angle and the target is harder side angle), compared to the other two difficulty conditions. This suggests that there is minimal benefit from seeing a more frontal prime beforehand—a harder side view is almost always disruptive and must be rotated to map onto an existing frontal representation. We interpret these results as evidence that seeing a more frontal view initially does not activate side views, and when later confronted with a side view at target, signers must then first rotate them to a frontal viewing angle to successfully process them, hence the additional processing cost. In the Easier angle condition (where the prime is a harder side angle and the target is easier/more frontal), rotation is already performed during the initial prime presentation. Thus, the more frontal Easier target can be processed more quickly, allowing for a larger priming effect between prime and target.

There are two independent pieces of evidence suggesting that mental rotation is involved in sign recognition from side angles. The first is that reaction times in the distance priming experiment correlate with mental rotation ability. The second is the linear increases in RT and error rates as angle moves further from 0° in the first half of the distance priming experiment (cf. the gradient prediction of Brozdowski and colleagues^55^, for processing classifier constructions from different viewing angles). This rotation helps the viewer to match phonological features to a representation in their mental lexicon. This is important because during ‘normal’ viewing conditions, i.e., face-to-face perception, a lot of phonological information is available early on in sign articulation, which helps limit possibilities during lexical retrieval^83^ From side angles, however, it is plausible that occlusions causing reduced phonological input impede and/or slow down recognition of the target sign because they leave more lexical options open for longer. Therefore, to facilitate sign recognition when adverse viewing angles cause occlusions, it is important to be able to quickly mentally rotate side-view input to activate stored frontal representations of signs. Our results also tie in with findings on mental rotation in signing populations which suggest that rotation ability improves in line with increasing fluency^60^. Indeed, it may be that signers’ mental rotation ability is better because of the frequent rotation required during signed language use in a variety of everyday deaf community settings.

A final result worth highlighting has to do with the main effect of Sign Type on processing speed. To our knowledge, the present study is the first to indicate a difference in comprehension by Sign Type, regardless of angle. We provide evidence that signs which are phonologically more complex, i.e. two-handed asymmetrical signs, take longer to process accurately, compared to less complex signs, such as one-handed signs. We had additionally hypothesised that Sign Type might interact with viewing angle, however this result was not significant. Nevertheless, there was a numerical trend whereby processing speed was slower at side angles for two-handed symmetrical signs (see Fig. 7b). This is in line with our predictions, since viewing a 2HS sign from a 90° angle entails a reduction in phonological input due to partial occlusion of one hand by the other. Xavier and Barbosa^84^ discuss a range of production modifications of two-handed Libras signs when one hand is unavailable, e.g. adding non-manual features or using a one-handed synonym, instead of simply articulating a two-handed sign with one hand only. Unlike our sign model in the lab, signers in the real world may use similar production modification strategies to accommodate, if they are aware that one of their hands is being occluded for those viewing from side angles.

Since we saw similar levels of priming in the Same and Easier conditions, our results differ here from Corina and colleagues^45^, whose repetition priming design saw more priming for same-angle (‘Front > Front’) prime-target pairs than ‘easier’ different-angle pairs (‘Left > Front’ or ‘Right > Front’). This difference may be due to our semantic categorisation task engaging deeper linguistic processing, instead of a visual-only task, such as distinguishing a gesture from a conventional sign. Since our semantic decision task required access to sign phonology to get to the meaning, our participants were likely relying on their linguistic representations much more than in a visual identification task. These linguistic representations had to be activated to process the harder prime trial initially, allowing for a greater priming benefit when seeing an Easier angle target trial, despite the many intervening trials in our distance priming design. Results in the Harder condition are similar to Corina and colleagues^45^, who saw greater priming for same-angle (‘Front > Front’) prime-target pairs than ‘harder’ different-angle pairs (‘Front > Left’ or ‘Front > Right’). Our present study showed larger differences in priming between Harder angle and Same angle targets, which is likely due to our distance priming design, with many trials between prime and target.

An important open question is the specific type of rotation required to process side views of signs, i.e. do viewers rotate to an egocentric perspective (a self-view of signing) or an allocentric perspective (viewing another person signing face-on)? While this study does not explicitly answer this question, we suggest that this rotation is likely to be to an allocentric front view. This is different to the 180° rotation required to map spatial indexing (i.e. locating grammatical referents in signing space) onto an egocentric view of the perceiver’s own body during face-to-face comprehension^51,55^. Here, we assume that rotation to an egocentric perspective is required only to facilitate comprehension of topographic spatial relations (i.e. when real-world spatial relations are depicted within signing space), which is not the case for our individual lexical signs. Given that the front viewing angle is most frequently encountered when signing, and there is relatively little visual self-monitoring during sign production, it would be highly inefficient to process all signed language input by rotating it to an egocentric view to map it on to your own body. However, there may be a role for egocentric representations or motor activation in particularly difficult processing conditions (e.g.^85,86^ but see also^87^).

As stated earlier, side viewing conditions are rarely encountered in L2 training materials, and though they do occur frequently in everyday interactions, signers often respond to them as they arise by reconfiguring their positioning. Given this clear preference for frontal viewing angles, one alternative explanation of our findings is that instead of a processing cost due to rotation, frequency is responsible for the adverse effect in semantic comprehension. However, real-world signing behaviours suggest that frequency is not driving this effect. Specifically, under naturalistic conditions, signers are likely to accommodate viewers from non-frontal perspectives by turning to face each other where possible. Additionally, as discussed in the section on proxemics and conversation circles, when signers are aware that they are signing to a large group or are trying to communicate despite suboptimal seating arrangements (e.g. a signer driving a car), they are likely to modify their production^18^. We would not expect signers to change their behaviours to account for frequency alone. Instead, we believe signers reconfigure positioning to make communication less cognitively challenging for all.

Furthermore, if there were a long-term ‘real-life’ frequency effect, we might expect it to disappear in a counterbalanced experiment, where it is mediated by experimental frequency. Indeed, the ‘inverse priming effect’, i.e. where less frequent primes have a greater priming effect compared to more frequent primes, has been found in production^88,89^ and in comprehension^90^. The latter study found that structural priming in Chinese sentence comprehension interacted with the frequency of alternative structures, whereby a less frequent structure was more sensitive to structural priming effects during self-paced reading. Our present study attempted to counterbalance the presentation of five different angles (0°, 45°R, 45°L, 90°R and 90°L), with the result that the two 45° and two 90° angles were each presented more frequently than the single 0° angle overall. Considering the inverse priming effect and the greater experimental frequency of non-frontal angles, we would have expected that a long-term frequency effect of angle would disappear within our experiment (see supplementary scripts). Since this is not what we found, we conclude that frequency *alone* cannot explain signers’ linguistic processing when confronted with a side viewing angle of a sign.

Crucially, while we believe the challenges of occlusions and the spatial transformation required to process signed input from side angles play a greater role than frequency, we cannot completely discount the possibility that frequency plays some part in processing signs from a side angle. Indeed, there is a well-known correlation between cognitive effort and frequency (e.g.^91^ whereby lower frequency words require more processing capacity, which could also be contributing to our results.

### Differences in L1 and L2 sign processing from side angles

Importantly, we found evidence that less fluent signers make disproportionately more errors when making semantic decisions to signs presented from side angles. Therefore, it may be helpful for signed language teachers and hearing L2 learners to increase learner exposure to difficult viewing conditions, by practising more group discussions and entering social situations where signs will be seen from multiple angles, instead of a single front-onsource^92^. Furthermore, it may be helpful for learners to expose themselves to signed language input that is as diverse as possible in domains beyond viewing angle, e.g. regional variation, handedness etc. If L2 signed language learners are aware of which viewing conditions of signed language input are most difficult, and which ones are most likely to benefit their learning, they may be more likely to push themselves to practise signing more in deaf spaces and get involved in group discussions.

The L2 Intermediate group showed by far the most variation in fluency as measured by the BSL-SRT, and it is the least fluent signers within this group who were most responsible for the disproportionate effect of angle. We predict that if the least fluent participants in the present study were to progress with their BSL learning journey, side angles would cease to be disproportionately difficult as their rotation ability improves and feeds back into their signing. Further research here could investigate the deliberate use of angle-diverse input and/or explicit mental rotation training from the very beginning of signed language learning, as well as tracking the development of mental rotation over the course of L2 signed language acquisition. We note that our four group sizes were relatively small, and while this is common in signed language research, studies focussing on diverse input in larger groups of L2 learners would provide greater statistical power. Dichotomising L2 signed language learners into two groups based on BSL qualifications alone is not an optimal approach (see e.g.^93^ for discussion of recent BSL qualification issues), especially since learners exhibit a broad range of fluencies, as we saw from the BSL-SRT. Thus it was valuable to also have this more granular assessment of fluency, which revealed results that the group analyses alone did not.

Although we only studied signs produced carefully in isolation, an important future step for research would be to understand L1 and L2 sign language processing in casual everyday conversations^94^. At the sentence level, factors such as disambiguation from sentential context^95^, phonetic reduction due to faster articulation^96^, and eye gaze shifts between different interlocutors etc., may all vary the adverse effect of side angles. Another next step for further research could be to use eye-tracking when processing a signed dialogue from the side. This could be informative regarding the processing of turn-taking in signed languages, an area which has not been investigated in much depth, particularly in L2 signed language learners. Furthermore, signers may follow different gaze fixation patterns under different viewing conditions. From a side angle, the neutral signing space in front of the body is further in the visual periphery, and the face, where signers usually fixate, is less clearly visible.

### Are side views of signs always harder?

Our questions dealt with angle-diverse processing across all 240 signs in the experiment. For most signs, the front angle was processed faster and more accurately than the 45° or 90° viewing angles. However, a post-hoc look at individual signs reveals that some were in fact easier to process from side angles than from the front, e.g. BEAR, BOTTLE, MATCH, MILK, NUT, RICE. In the supplementary scripts we make available a breakdown of RT and accuracy results from each angle for the signs featured in this experiment, in the hope this could be informative for BSL teachers and L2 signed language learners. Deaf signers and signed language teachers do seem to be aware of the poorer visibility of certain signs from frontal viewing angles, shifting their bodies to show signs with forward–backward movement from a 45° viewing angle, particularly when using 2D video conferencing such as Zoom (e.g. FUTURE in ASL^97^; Skyer, personal communication). Some teaching resources, both online and offline, also display signs from a front and a 45° angle. Relatedly, several facial recognition studies report better in-person recognition at 45°^80,98,99^. Furthermore, several of the deaf participants in Peterson^100^ self-reported a preference for a slightly offset 30° angle over 0°, 45° and 60° while watching simultaneous speech and sign. According to participants and comments from deaf professionals, non-frontal angles give an “illusion of a third dimension which seemed to make the task of reading [comprehending] the televised signs and fingerspelling easier” [^100^﻿, p. 47]. However, non-frontal angle preferences in digital domains may be partly driven by the lack of binocular depth cues, available in-person but not through screens.

While we did not see any overall advantages for processing signs from 45° viewing angles in the present study, a frontal 0° angle may not always be best for learning to produce new signs, or for processing a signed language at the sentence level. Sign language teachers working online during the COVID-19 pandemic also specifically name the frontal viewing angle and the difficulty of turning and repositioning their bodies on camera as one of the hardest aspects of teaching signed languages through a two-dimensional screen^101^. For situations like these, where the three-dimensional nature of signed language is inhibited, it is possible that side or slightly offset viewing angles may even be a beneficial rather than an adverse viewing condition.

## Conclusion

The results presented here provide evidence for the nature of signed language processing and how signs may be represented and accessed in the brain. Specifically, we found evidence that viewing angle is an important consideration for comprehending individual BSL signs, such that there was a significant gradient increase in reaction time and decrease in accuracy from 0° to 45° to 90° in turn. These results suggest that existing representations of signs in the mental lexicon are stored first and foremost from a frontal view and that viewing other angles incurs a processing cost. Furthermore, for less fluent hearing L2 signed language learners, we saw disproportionately more errors to signs seen at side angles when compared to more fluent signers. As a result, we recommend greater consideration of learner exposure to various viewing angles in signed language teaching contexts, as an aspect of modality-focussed L2 signed language instruction^102^. Improvements in mental rotation ability as sign language fluency increases^60^ should also be paid attention to, since better mental rotation ability seems to enable faster and more accurate rotation of lexical representations of signs, thus mitigating the adverse effect of side viewing angles. More generally, extralinguistic viewing conditions for signed language processing should be considered to the same detailed extent that adverse listening conditions have been studied for speech perception, both to improve learner outcomes and to discover more about the linguistic processing of signed languages.

## Data Availability

In line with standards of reproducible research, the scripts and data are made available with this publication and can be retrieved on the following publicly accessible repository: https://osf.io/njmhb.
